# A prospective, randomized placebo‐controlled clinical trial on the effects of a fluoride rinse on white spot lesion development and bleeding in orthodontic patients

**DOI:** 10.1111/eos.12186

**Published:** 2015-04-25

**Authors:** Nicoline C. W. van der Kaaij, Monique H. van der Veen, Marleen A. E. van der Kaaij, Jacob M. ten Cate

**Affiliations:** ^1^ Department of OrthodonticsAcademic Centre for Dentistry Amsterdam (ACTA)University of Amsterdam and Free University AmsterdamAmsterdamthe Netherlands; ^2^ Department of Preventive DentistryAcademic Centre for Dentistry Amsterdam (ACTA)University of Amsterdam and Free University AmsterdamAmsterdamthe Netherlands; ^3^ Department of Internal MedicineVU University Medical CentreAmsterdamthe Netherlands

**Keywords:** caries prevention, fixed orthodontic appliances, fluoride rinse, gingival bleeding, tooth demineralization

## Abstract

Demineralizations around orthodontic brackets are a main disadvantage of orthodontic treatment. Several methods have been advocated to prevent their development, such as fluoride rinses or varnishes. In this randomized clinical trial, a fluoride rinse (a combination of sodium fluoride and amine fluoride) was compared with a placebo rinse, to be used every evening after toothbrushing. A total of 81 participants (mean age: 13.3 yr) completed the study (mean treatment period: 24.5 months). Demineralizations, measured using quantitative light‐induced fluorescence and the decayed, missing, and filled surfaces (DMFS) index, were assessed before treatment (baseline) and around 6 wk after debonding (post treatment). Bleeding scores were measured at baseline, and during and post treatment. The incidence rate ratio for demineralizations was 2.6 (95% CI: 1.1–6.3) in the placebo group vs. the fluoride group. In the fluoride group, 31% of participants developed at least one demineralization, compared with 47% in the placebo group. Relative to baseline, gingival bleeding increased significantly in the placebo group 1 yr after the start of treatment and onwards. For the fluoride group, bleeding scores during treatment were not different from those at baseline. In conclusion, using a fluoride rinse helps to maintain better oral health during fixed appliance treatment, resulting in fewer demineralizations.

Most orthodontic patients are treated for aesthetic reasons, with only a small number of patients receiving orthodontic treatment for medical or dental indications 1. Any potential disadvantages, such as demineralizations, must therefore be taken into account before treatment starts. The environment in the oral cavity of adolescents will be affected by the placement of fixed orthodontic appliances, through changing the microbial composition and increasing the number of retention sites and thus plaque formation 2, 3. In turn, this disturbance of a balanced microbial ecology may contribute to oral diseases, such as caries 4 and periodontitis 5, 6. Because clinical investigations have shown that generalized gingivitis develops within 1–2 months of placement of fixed appliances 7, good oral hygiene is an important prerequisite for sustaining oral health during orthodontic treatment 8, 9 and also for preventing the formation of white spot lesions (WSL) in enamel 10. According to the literature, the prevalence of WSL ranges from 50% to 97% 11, 12, 13, 14, depending on the examination technique used and the duration of treatment.

Clinical studies have used several methods to detect and measure WSL, based on clinical indices 11, photographic examinations 15 or on other optical methods, such as quantitative light‐induced fluorescence (QLF) 12, 16. Quantitative light‐induced fluorescence is an optical, visible light‐based system that can be used to detect and quantify early demineralization of enamel.

Various methods of reducing the formation of WSL have been described, including improvement of oral hygiene and the use of additional fluoride, such as in varnishes or rinses. The most common oral hygiene protocol recommended by orthodontists is probably a daily 0.05% sodium fluoride rinse in conjunction with fluoridated toothpaste 17. However, although this recommendation is based on research showing that the use of sodium fluoride rinse significantly reduces caries rates in non‐orthodontic patients, the evidence with regard to its efficacy in preventing WSL in orthodontic patients is inconclusive 18. Some moderate evidence shows that fluoride varnish applied every 6 wk during orthodontic treatment is effective 18, 19.

In this randomized clinical trial (RCT) we compared the ability of a fluoride rinse (a combination of sodium fluoride and amine fluoride) with a placebo rinse to prevent WSL formation in patients undergoing orthodontic treatment with fixed appliances. The incidence of WSL was assessed using QLF.

## Material and methods

### Study population and clinical procedures

An RCT was performed, under normal practice settings, to determine the efficacy of a fluoride rinse during orthodontic treatment with fixed appliances. Approval of the Medical Ethical Committee of the VU Medical Centre of the VU University of Amsterdam was obtained for this study (VU‐METc 2009/026 and Dutch trial register: NTR1817).

The study was conducted at the Department of Orthodontics of the Academic Centre for Dentistry Amsterdam (ACTA). Patients who were scheduled for placement of full fixed orthodontic appliances at ACTA were eligible to participate after providing written informed consent. Patients needed to fulfil the following inclusion criteria: (i) 10–18 yr of age; (ii) good general health; (iii) no use of medication; and (iv) no demineralizations in need of restoration present at a buccal surface. All patients selected for this study received fixed appliances in both jaws. The brackets used were Roth Ovation Brackets (Dentsply GAC International, Bohemia, NY, USA). After placement of the fixed appliances the participants were randomly assigned to rinse either with solution A, which contained 250 ppm fluoride (100 ppm amine fluoride and 150 ppm sodium fluoride) (Elmex caries protection; Colgate‐Palmolive Europe, Therwil, Switzerland), or solution B, which was a fluoride‐free placebo rinse (also provided by Colgate‐Palmolive Europe); solution A and solution B are subsequently referred to as fluoride rinse and placebo rinse, respectively. The bottles containing the fluoride rinse and the placebo were identical in appearance, consistency, taste, and smell. This was tested and regulated by Colgate‐Palmolive Europe. Allocation of participant id was determined according to order of inclusion and appointment scheduled by reception staff. Assignment of participants to a study group was made at the first appointment by using a predefined randomization list (made in Microsoft Office Excel 2003). Participants were informed that they would receive either a rinse containing fluoride or a placebo rinse. Participants, examiners, statisticians, and orthodontic postgraduates delivering the treatment were blinded for test and placebo product type. During the study period, participants were instructed not to use any fluoride‐containing products other than fluoride toothpaste. The participants' dentist was informed about the ongoing study and was instructed not to apply extra fluoride during the study period. All examinations were performed mainly by the researcher (N.K.) and by trained dental students. Approximately 1 wk before placement of the fixed appliances (T0), QLF images were made and an intra‐oral examination was performed. Quantitative light‐induced fluorescence images of buccal surfaces of all teeth in upper and lower jaws from the second premolar to the second premolar were captured. Participants were clinically examined using the decayed, missing, or filled surfaces (DMFS) index 20 and the International Caries Detection and Assessment System (ICDAS) 21, 22, 23, followed by assessment of gingival bleeding. Participants were assessed, at regular intervals [approximately 6 wk (T1), 12 wk (T2), 6 months (T3), and every 6 months thereafter during treatment (T4 and up)] during the orthodontic treatment, to stimulate optimal oral hygiene, to supply the rinse, and to look for unwanted signs of developing caries. Bleeding was also recorded at these time points. At the day of debonding (TD) and around 6 (TD1) and 12 (TD2) wk after debonding, DMFS, ICDAS, and bleeding scores were assessed, and QLF images were made to quantify the WSL. The caries assessments of TD1 were used for data analyses.

**Figure 1:**
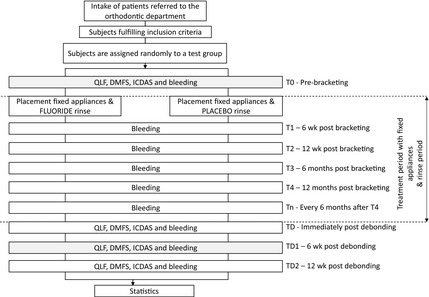
Flow‐chart showing the different study time points and corresponding assessments. The caries assessments made at TD1 were used for the analyses. ICDAS, International Caries Detection and Assessment System; DMFS, decayed, missing, and filled surfaces; QLF, quantitative light‐induced fluorescence.

Figure 1 shows a flow chart with the different time points and measurements. End of data collection was set at January 2013. After analysing all data obtained, the code regarding the rinse was broken.

### Study parameters

The primary study parameter was the number of caries WSL, as found by QLF, that developed during treatment with fixed orthodontic appliances.

Secondary study parameters were: the ICDAS score and DMFS index measured before and after treatment with fixed orthodontic appliances; the bleeding scores per participant measured at different time points during treatment with fixed orthodontic appliances; and the extent of the WSL as determined by QLF (fluorescence loss and lesion area) after debonding.

### Power analysis

No such studies performed previously are known. A power analysis was performed for an effect of 0.25 (with a power of 0.8 and a significance level of 0.05). This resulted in a total of 94 participants or 47 participants per study group. To compensate for participant attrition, we aimed to include 120 patients in total.

### QLF imaging; WSL measurements

Fluorescence images of the (to be) bonded buccal surfaces were captured using an intra‐oral fluorescence camera (QLF/Clin; Inspektor Research Systems, Amsterdam, the Netherlands) 24, 25. Dedicated software (inspector‐pro version 2.0.0.48; Inspektor Research Systems) was used to assess the QLF images after debonding (fluorescence loss, i.e. white spots). Quantitative light‐induced fluorescence images were analysed for fluorescence loss (ΔF) and size of lesion area using a threshold of 5%, at TD1 in comparison with the QLF images made at T0. If caries was present at T0, the results were subtracted from TD1, using the method described by mattousch *et al*. 16. The number of lesions per participant was calculated and, for every participant having at least one lesion, mean ΔF and area were calculated.

The measurements were carried out by the same examiner (N.K.). The examiner (N.K.) was trained and calibrated for QLF assessments against an experienced examiner (M.V.) before the study start. Inter‐ and intra‐observer reliability were established at a random sample of 10% of the participants with an interval of 2 wk. The inter‐examiner intraclass correlation coefficient (ICC) scores for QLF were 0.92 for the ΔF and 0.96 for the lesion area. The intra‐examiner ICC was 0.94 for ΔF and 0.98 for the lesion area.

### DMFS

The DMFS of all participants was scored by examining all teeth with the use of a mouth mirror, an explorer, and optimal light 20. Also, radiographs (orthopantomographs and, when available, bitewings or solo‐images) were checked carefully. The D‐portion comprised all surfaces with signs of decay diagnosed clinically as caries lesion with enamel breakdown. To determine the M‐portion of the DMFS, only surfaces missing because of caries were counted. Teeth extracted for orthodontic purposes were not included. Restorations made because of trauma were excluded from the F‐portion.

### ICDAS

Before placement (T0), and after removal of the appliances (TD, TD1, and TD2), the buccal surfaces from all bonded teeth were examined using the ICDAS assessment system 21, 22, 23. Each buccal surface was given a code from 0 to 6 to express the degree of caries: code 0 was given for a sound surface that was unchanged after air‐drying, except for stain, hypoplasia, wear, erosion, and other non‐caries phenomena; code 1 was given for the first visual change in enamel, seen after air‐drying; code 2 was given if a distinct visual change (white or coloured) was seen on the wet enamel surface; code 3 was given when local enamel breakdown was present, but without visible dentine; and codes 4 to 6 were given to cavitated lesions with increasing severity. Average ICDAS‐scores were calculated for each participant.

### Bleeding score

During each visit, a gingival bleeding score was determined by probing each (to be bonded or bonded) tooth mesiobuccally and distobuccally with a periodontal probe. Based on the percentage of sites with bleeding, bleeding scores of 1 to 5 were given for the whole mouth (and thus per participant). Score 1 (good) was given if 0–5% of the sites were bleeding; score 2 (medium/good) if 6–10% of the sites were bleeding; score 3 (medium) if 11–20% of the sites were bleeding; score 4 (medium/poor) if 21–35% of the sites were bleeding; and score 5 (poor) if >35% of the sites were bleeding.

### Statistical analyses

Statistical analyses were performed using ibm spss 20.0 and stata (intercooled stata 10.0; Stata, College Station TX, USA).

We estimated the difference in number of WSL (primary end‐point) and DMFS index between participants with fluoride rinses and those with placebo rinses, using a regression model. Because both number of WSL and DMFS index are count variables, and our data were over‐dispersed (variance much greater than mean), we used negative binomial regression 26, 27. Likelihood ratio tests comparing our negative binomial models with Poisson regression models were used to evaluate our decision. Because caries lesion data often exhibit an excess number of zeroes 28, we also compared our negative binomial models with zero inflated negative binomial regression models using the Vuong test. Both tests indicated that a negative binomial regression model offered the best possible fit. Results were expressed using the estimated incidence rate ratio (IRR) and 95% CI were constructed.

To investigate possible confounding by treatment duration, bleeding, DFMS, or ICDAS scores at T0, we added these parameters to the model. None induced a change of more than 10% in the estimated IRR, so no confounding was present.

Differences in ICDAS, fluorescence loss, and lesion area were tested using the Mann–Whitney *U*‐test. The Wilcoxon signed‐rank test was used to compare the bleeding score at different time points, with a Bonferroni correction for multiple comparisons.

## Results

### Descriptive results

**Figure 2:**
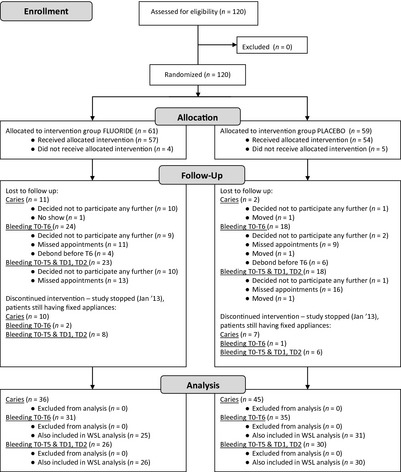
Flow‐chart showing the participant follow up during the study and the number of participants available for each analysis. Treatment periods with appliances: T0, prebracketing; T1, 6 wk post bracketing; T2, 12 wk post bracketing; T3, 6 months post bracketing; T4, 12 months post bracketing; T5, 18 months post bracketing; and T6, 24 months post bracketing. Caries assessment time points: TD, immediately post bracketing; TD1, 6 wk post bracketing; and TD2, 12 wk post bracketing. WSL, white spot lesion.

A total of 120 participants were entered into the study between April 2009 and January 2011. Nine participants declined further participation immediately after T0 (approximately 1 wk before bonding), at placement of the fixed appliances, and therefore did not receive the allocated rinse. A further 11 participants declined to participate later during the study, in addition to one participant who moved away and one who failed to show up for appointments. The fixed appliances of 81 of the 98 remaining participants were debonded before January 2013. This point was chosen to end the study according to protocol. At the study end‐point, 17 participants were expected to continue treatment with fixed appliances for more than 3 months, exceeding the study period, as a result of unforeseen treatment complications or non‐compliance. The mean ± SD treatment time was 24.5 ± 5.5 months. A flow‐chart for all different analyses is shown in Fig. 2.

Table 1 contains baseline data; there were no significant differences between groups. There were no WSL present at baseline. Caries data 6 wk after debonding (TD1) were used for 77 participants. For three, who missed appointment TD1, the WSL assessments (QLF, DMFS, and ICDAS) from immediately post‐debonding (TD) were used, and, for one participant, the QLF pictures were only made at TD2 because of malfunction of the QLF‐device. The WSL assessments were made at an average of 52 d after debonding (with a range of 0–156 d).

**Table 1:** Characteristics of the study group at baseline (T0)

Characteristic	Fluoride (*n* = 36)	Placebo (*n* = 45)	All (*n* = 81)
Age (yr)*	13.1 (10.0–16.6)	13.6 (11.7–16.5)	13.3 (10.0–16.6)
Male gender, *n* (%)	14 (38.9)	21 (46.7)	35 (43.2)
Treatment duration (months)*	25.0 (12.0–36.3)	24.1 (13.3–37.6)	24.5 (12.0–37.6)
DMFS score***			
0	67	58	62
1–2	11	24	19
3–4	19	7	12
≥5	3	4	7
DMFS score overall**	0 (14)	0 (13)	0 (14)
ICDAS***			
0.00	47	53	51
0.01–0.05	17	11	14
0.06–0.15	17	18	17
≥0.16	20	18	18
ICDAS score overall**	0.05 (0.8)	0 (0.7)	0 (0.8)
Bleeding*	2 (1–4)	1 (1–3)	1 (1–4)

Data are expressed as *mean (range), **median (maximum), or ***percentage.

ICDAS, International Caries Detection and Assessment System; DMFS, decayed, missing, and filled surfaces.

Given the difference in treatment duration between participants, and taking account of the loss to follow‐up, the bleeding data were assessed in two separate steps: participants having a complete data set from baseline until T6 and participants having a complete data set from baseline until T5, TD1, and TD2. A total of 66 participants (31 in the fluoride group and 35 in the placebo group) were analysed who had a complete data set regarding bleeding from the start (T0) until 24 months after the placement of fixed appliances (T6; mean: 727 d). Of these participants, 25 who received fluoride rinse and 31 who received placebo rinse were also analysed for WSL after debonding. Fifty‐six participants (26 in the fluoride group and 30 in the placebo group) were analysed with a complete data set regarding bleeding from the start (T0) until 18 months after the placement of fixed appliances (T5; mean: 555 d) and TD1, 6 wk after debonding (mean: 50 d) and TD2, 12 wk after debonding (mean: 99 d). As many appliances were removed between T5 and T6, T6 was excluded from this analysis.

### QLF results

**Figure 3:**
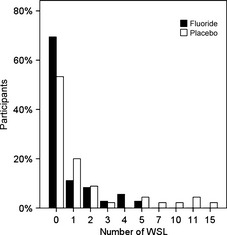
Percentage of total participants and their white spot lesion counts, 52 d after debonding. In the fluoride group, 69.4% of the participants were WSL‐free, compared with 53.3% in the placebo group after treatment with fixed orthodontic appliances.

Of the 81 participants, 32 had developed at least one WSL (39.5%). In the fluoride group, 11 of 36 participants developed at least one WSL (30.6%), ranging from one to five WSL per participant (Fig. 3). In the placebo group, 21 of 45 participants had at least one WSL (46.7%), with a range of one to 15 WSL per participant. Participants in the placebo group had an IRR of 2.6 (95% CI: 1.1–6.3) compared with the fluoride rinse group (*P *=* *0.038). The DMFS index, ICDAS, and bleeding at T0 and treatment duration were not confounders.

The mean ΔF (%) and mean lesion area (mm^2^) was calculated for each participant with at least one WSL (fluoride group, *n* = 11; and placebo group, *n* = 21). The mean ± SD ΔF was 10.3 ± 3.0% for placebo participants and 11.6 ± 5.0% for fluoride participants. Mean ± SD lesion area was 1.3 ± 1.6 mm^2^ for placebo participants and 0.9 ± 0.6 mm^2^ for fluoride participants. At a mean of 52 d after debonding there were no statistically significant differences in mean ΔF and mean lesion area per participant between both groups (Table 2).

**Table 2:** Results, 6 wk post debonding (TD1), according to study group and for the total group

Variable	Fluoride		Placebo		All	
	*n*	TD1	*n*	TD1	*n*	TD1
WSL						
Number	36	0 (5)*	45	0 (15)*	81	0 (15)
Mean ΔF (%)	11	11.6 ± 5.0	21	10.3 ± 3.0	32	10.7 ± 3.8
Mean A (mm^2^)	11	0.9 ± 0.6	21	1.3 ± 1.6	32	1.15 ± 1.4
DMFS	36	0 (26)	45	1 (13)	81	1 (26)
ICDAS	36	0.05 (0.6)	45	0.05 (1.7)	81	0.05 (1.7)

Data are expressed as median (maximum), or as mean ± SD.

ΔF, fluorescence loss; A, size of lesion area; DMFS, decayed, missing, and filled surfaces; ICDAS, International Caries Detection and Assessment System; WSL, white spot lesions.

*Significant difference (*P *<* *0.05) between fluoride and placebo groups.

### DMFS results

The DFMS scores at TD1 ranged from 0 to 13 in the placebo group and from 0 to 26 in the fluoride group (Table 2). In the fluoride group there was one outlier; this subject started with a DMFS score of 14 and, after debonding, had a DMFS score of 26. Both groups showed a significant increase in DMFS between T0 and TD1. A negative binominal regression analysis showed that there were no differences in the DMFS scores between both groups.

### ICDAS results

There were no significant differences between the placebo and fluoride rinses regarding the ICDAS scores after debonding. Also, no significant differences were found between T0 and TD1 regarding the ICDAS scores (fluoride, *P *=* *0.88; and placebo, *P *=* *0.06) (Table 2).

### Bleeding

#### Bleeding T0‐T6

**Figure 4:**
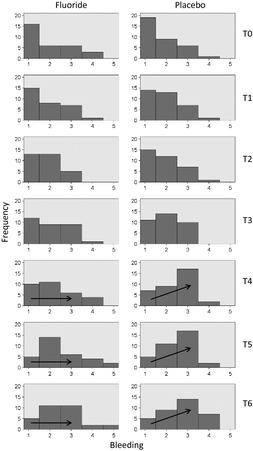
Bleeding scores during visit T0 (prebracketing) and visits T1–T6 (6, 12 wk, 6, 12, 18, and 24 months post bracketing), for the fluoride (left panel) and placebo rinse (right panel). At visit T4 and at subsequent visits the placebo group differs significant compared to baseline. The fluoride group did not show a difference.

Gingival bleeding scores of the individuals receiving the placebo rinse were significantly higher at three points compared with the respective baseline visit scores: visit T4, 12 months (mean = 373 d) since the start of treatment (*P *=* *0.02); visit T5, 18 months (mean = 546 d) since the start of treatment (*P *=* *0.00); and visit T6, 24 months (mean = 718 d) since the start of treatment (*P *=* *0.00). For the group receiving the fluoride rinse, this difference did not reach statistical significance (Fig. 4). A Mann–Whitney *U*‐test showed no differences between the groups at the different time points.

#### Bleeding T0‐T5 and TD1, TD2

Comparison of participants in the two groups indicated that there were no differences after removal of the fixed appliances.

## Discussion

This is the first randomized, triple‐blind, placebo‐controlled study showing that a fluoride rinse reduces the formation of WSL during treatment with fixed orthodontic appliances. Participants using placebo developed 2.6 times more WSL during the study period compared with participants who used a daily fluoride rinse containing 100 ppm amine fluoride and 150 ppm sodium fluoride. In the fluoride group, 31% of participants developed at least one WSL. A previous study showed that 33.5% of the patients developed WSL after using a 0.05% sodium fluoride rinse; no placebo was used in that study 29. In our study, 47% of the participants developed at least one WSL whilst using a placebo rinse. This figure is comparable with those in the literature, which report WSL development in around 50% of subjects without a preventive method or after using a placebo foam 11, 30. We could not demonstrate a reduction in overall lesion size and depth using a fluoride rinse if WSL developed. Our finding, of considerably fewer WSL than observed in the QLF study of boersma *et al*. 12, might be because the ACTA orthodontic department has introduced a stringent oral hygiene protocol since that study was carried out.

In the present study we measured the number of WSL (using QLF, DMFS, and ICDAS) 6 wk after debonding, as it is known that the gingival swelling immediately after debonding obscures part of the buccal surfaces, but recedes 6 wk later, thus showing a higher number of WSL at TD1 12.

White spot lesions are not only an aesthetic problem; after debonding, an overall improvement is seen in only 36% of the lesions, a large number (49%) of the caries lesions remain stable over time, and 15% of lesions are in need of, or received, restorative care 2 yr after debonding 16.

Compliance is often mentioned as a shortcoming of prescribing a rinse. It has been reported that the more compliant a patient, the fewer WSL that are formed 29. geiger *et al*. 29 also found that those patients who exhibited poor oral hygiene, but were strict in their rinsing, showed a reduction in the incidence of WSL. As both groups in our study used a rinse, we assume that compliance was similar in both groups. Compliance was not checked, thus mimicking normal practice settings. Thus, we demonstrated that a rinse is an effective method for WSL prevention during treatment with fixed orthodontic appliances, even if participants may be non‐ or partially compliant.

Even though we showed a difference in WSL development between groups, we did not meet the goal of our power analysis: first, because of the overall load of the appointments, although mostly scheduled in combination with regular visits, resulted in a higher attrition; and, second, extension of treatment duration in combination with the end‐point of January 2013 resulted in fewer participants in our analyses than planned.

Our study showed that rinsing with fluoride helped to maintain good oral health during treatment with fixed orthodontic appliances, as evidenced by a lower bleeding score over time. For non‐orthodontic populations it is known that use of a Meridol (amine/stannous fluoride) (Gaba International, Therwil, Switzerland) rinse retards the development of gingivitis, resulting in a lower bleeding score as well as a lower plaque gingival bleeding indice 31, 32. In an orthodontic population, one study reported the effect of Meridol on bleeding 33, indicating that bleeding increased significantly between bonding and debonding for the group only using a fluoride toothpaste, whereas in the group using Meridol, there was no change in bleeding between bonding and debonding. No other studies are known that show a positive effect on bleeding using a fluoride rinse during fixed appliance treatment.

Based on this study, we conclude that the prescription of a fluoride rinse, to be used at home, has a measurable preventive effect on overall oral health. It helps to prevent WSL formation and lessen the number of WSL formed and maintains better gingival health (measured as bleeding).

## Conflicts of interest

This study was supported by Elmex research/Colgate‐Palmolive Europe, Therwil, Switzerland. Both the placebo and the fluoride rinse were supplied by Elmex research/Colgate‐Palmolive Europe, Therwill, Switzerland. Dr M. H. van der Veen is a co‐inventor of several patents relating to quantitative light‐induced fluorescence. The authors declare that otherwise there is no conflict of interest pertaining to the data presented in this article.
